# Comparison of hospital charge prediction models for gastric cancer patients: neural network vs. decision tree models

**DOI:** 10.1186/1472-6963-9-161

**Published:** 2009-09-14

**Authors:** Jing Wang, Man Li, Yun-tao Hu, Yu Zhu

**Affiliations:** 1 Department of Epidemiology and Biostatistics, School of Public Health, Anhui Medical University, Anhui province, PR China; 2 Department of Medical Information, the First Affiliated Hospital, Anhui Medical University, Anhui province, PR China; 3 Department of Clinical Medicine, Anhui Medical University, Anhui province, PR China; 4 Department of Epidemiology and Biostatistics, School of Public Health, Anhui Medical University, Anhui province, PR China

## Abstract

**Background:**

In recent years, artificial neural network is advocated in modeling complex multivariable relationships due to its ability of fault tolerance; while decision tree of data mining technique was recommended because of its richness of classification arithmetic rules and appeal of visibility. The aim of our research was to compare the performance of ANN and decision tree models in predicting hospital charges on gastric cancer patients.

**Methods:**

Data about hospital charges on 1008 gastric cancer patients and related demographic information were collected from the First Affiliated Hospital of Anhui Medical University from 2005 to 2007 and preprocessed firstly to select pertinent input variables. Then artificial neural network (ANN) and decision tree models, using same hospital charge output variable and same input variables, were applied to compare the predictive abilities in terms of mean absolute errors and linear correlation coefficients for the training and test datasets. The transfer function in ANN model was sigmoid with 1 hidden layer and three hidden nodes.

**Results:**

After preprocess of the data, 12 variables were selected and used as input variables in two types of models. For both the training dataset and the test dataset, mean absolute errors of ANN model were lower than those of decision tree model (1819.197 vs. 2782.423, 1162.279 vs. 3424.608) and linear correlation coefficients of the former model were higher than those of the latter (0.955 vs. 0.866, 0.987 vs. 0.806). The predictive ability and adaptive capacity of ANN model were better than those of decision tree model.

**Conclusion:**

ANN model performed better in predicting hospital charges of gastric cancer patients of China than did decision tree model.

## Background

Gastric cancers form the leading cause of deaths. Ninety-five percent of gastric cancers are adenocarcinomas, derived from the epithelium [1]. Substantial geographic variations exist in the incidence of gastric carcinomas internationally and they consist the most common cancers in China [2]. With changing diet and life style and the development of new health policies, more and more gastric cancer patients have been discovered in routine medical check ups. Consequently, total hospital charge on gastric cancer patients and its composition is changing. Therefore, it is important to develop appropriate methodologies to model and predict hospital charges on gastric cancer patients and their relations with other factors.

Standard regression methods had been commonly applied in predicting hospital charges in previous studies [3-5]. However hospital charge data characterize non-normal distribution, existence of many related factors and substantial inter-actions between the related factors. These undermine the fundamentals of standard regression analysis. In recent years, uses of ANN models in the mining of complex information in medical fields have been increasing [6]. ANN is advantageous in that it has no particular requirements about data distribution and is fault tolerable. These make ANN most suitable in dealing with complex multi-variable relationships [7]. Examples of such applications include identification of prostate cancer, prediction of risk factors of coronary heart diseases, collocation of medicine dosage and so on [8]. Some researchers had found that the ANN models were more accurate in predicting hospital charges [9-12]. Although the nature of "black box" with ANN makes it difficult in interpreting modeled results, we had retrieved 28 articles from the literature documenting application of ANN in medical field. By comparing the prediction efficient of ANN model and standard regression methods, these studies all conluded that ANN model is superior to standard regression methods [13].

With regard to decision tree of data mining technique, it was recommended by some researchers because of its richness of classification arithmetic rules and appeal of visibility. In one study [14], Seung-Mi Lee compared the prediction efficient of ANN and decision tree on hospital charges on colon cancer patients. In other studies [15-18] researchers compared these two kinds of methods in other fields. Findings of these comparisons were mixed and researchers had not reached an agreement on whether ANN model is superior to decision tree model or vice vers.

This study aims at applying ANN and decision tree models to predict hospital charges on gastric cancer patients and comparing their predictive abilities, so as to shed new lights on methodology for the prediction of the hospital charge on gastric cancer patients.

## Methods

### 1. Human right protection

Our research was in compliance with the Helsinki Declaration [19] approved by the ethics committee of Anhui Medical University.

### 2. Data preprocessing

First, we performed variable selection with the help of medical experts and administrators of the hospital. For example, we were advised by the exports that "type of operations" is surely related to charges and that patient's "dwelling area" may reflect his or her income conditions, so we included these two variables. In addition, the original dataset contained variables with null values. For example, the dataset included 'emergency treatments _ 1' through to 'emergency treatment _10' used to record whether a patient had been rescued for up to ten times. However it is rare that a patient receives many times of emergency treatments during an admission period, which led to a lot of blanked fields. Therefore we created, 'emergency treatments' for storing the times of emergency treatments of each critical patient and 'being rescued' for storing times of the successful emergency treatments. We also performed variable selection by means of uni-variable analyses methods, such as t-test, chi-square test and analysis of variance with the inclusion criteria of 0.05. As a result, 12 variables were selected including age, sex, marriage, dwelling area, operation or not, type of operations, chemotherapy or not, radiotherapy treatment or not, emergency treatments, times of being rescued, length of stay (LOS) and ways of payment.

### 3. Artificial neural networks (ANN)

Neural networks, with neurons as the basic building blocks, were computer systems that attempt to model the way human brain works. A well-known method was a feed-forward back-propagation (BP) network, since the data used to train the network was presented at the first (input) layer and then feed forward through the hidden layer(s) to produce a response at the output layer. The connections between the artificial neurons were adjustable parameters with a sign and a magnitude and training involved adjustment of these connection strengths (weight) until some desired output (target) signals was produced. A common form of training involves starting the network with random values for the connection weights, presentation of the data and calculation of output signals. The output values were compared with the targets and the weights were adjusted backwards through the network to the input layer.

In this study, BP network was used and the input variables were the twelve factors selected above and the output variable was the hospital charge on gastric cancer patients. The transfer function of sigmoid was used in ANN model with 1 hidden layer and 3 hidden nodes. The training algorithm was f (x) = 1/[1+exp (-x)].

The sensitivity was calculated as follows: firstly, output value Y was translated into 0~1; according to a variable X, the value of each case in the sample was changed one by one (in which if the variable was category, all the possible category combinations were tested; if the variable was numerical, the minimum, lower quartile, median, upper quartile with the maximum values were tested); then, the system would note the output values of the maximum Y_max _and minimum Y_min _and calculate the proportion of (Y_max_-Y_min_)/Y_max_; the sensitivity of the variable X was the mean of proportions of all the cases. The accuracy was equal to Σ(1-|t_i_-o_i_|)/n, in which t_i _and o_i _expressed the measured value and predicted value respectively of the *i*th case and n was the number of sample.

### 4. Decision tree

Decision tree was a tree construction similar to control diagram, in which each inside node expressed the test of an input property with its branch representing the output of test and each branch node represented a category or its distribution. Recursion partition method was applied to make decision tree from top to bottom. When a decision tree was made, a new sample was categorized from the tree root node to a leaf node based on the values of all the properties with category rules. The arithmetic of classification and regression was commonly used. Rules are directly observable through decision tree induction; that is to say, the classification rules of the hospital charge on gastric cancer patients could be captured from the decision tree.

The data were split into training (70% of sample and 706 cases) and test (30% of sample, 302 cases) datasets by stratified sampling method. The hospital charge was used as the output variable with the 12 variables including age and sex etc as input variables. The fitness of BP ANN model and C&T decision tree model was analyzed using SPSS Clementine11.0. Mean absolute error and linear correlation coefficient were used to evaluate and compare the fitness strength. Moreover, sensitivity analysis in the fitness of BP ANN model was performed on these input variables, in order to estimate the relative importance of them. If the sensitivity of a variable was larger, its importance on the hospital charge was stronger.

## Results

The dataset was derived from the digitalized records of gastric cancer patients who had been treated in the First Affiliated Hospital of Anhui medical University from January 1^st ^of 2005 to December 31^st ^of 2007. A total of 1008 patients met our selection criteria, i.e. diagnosed by either radiography or endoscope as suffering from gastric cancers. 405 men and 603 women, aged from 22 to 85 years with an average age of 56.75 years. Their average length of stays was 11.36 days ranging from1 to 51 days. Among them, 20 patients were married and the other 988 unmarried. The number of patients came from counties, suburban and urban areas added up to 230, 478 and 300 respectively. 13 patients were allergic to penicillin and 3 patients were allergic to procaine. 573 patients were treated with various types of operations including subtotal gastrectomy, total gastrectomy, gastrectomy with extended lymphadenectomy and palliviate operations. 368 patients received chemotherapies and 8 patients underwent radiotherapy treatments. 3 patients had been rescued twice with success. 5 patients had been rescued once time and 2 survived. 190 patients paid the charge themselves and 818 patients were paid by public health insurance.

The median of the hospital charge on these 1008 gastric cancer patients was 13803.84 RMB with the lowest hospital charge of 149 RMB and the highest 70606.3 RMB. This sum of charges on gastric cancer patients consisted of medicine charge, operation charge, treatment charge, bed charge and other charges in which the highest proportion was the medicine charge followed by the treatment charge.

### 1. Univariate analyses

Some results of univariate analyses such as t-test and ANOVA were listed in table 1. At the same time, chi-square test was done between marriage situation and the ways of payment. Frequencies of private and public insurance in un-married and married persons were 178, 810, 10 and 10 respectively (χ^2 ^= 11.193, *P *< 0.05). Ages of men and women were 54.19 ± 11.86 and 58.43 ± 11.17 with statistically significance (t = 5.695, *P *< 0.05). Additionally, correlation among the total charge, age and LOS was performed to find that there existed correlation-ship. Coefficient of correlation (*r*) between the total charge and age was 0.159 with *P *< 0.05; *r *between the total charge and LOS was 0.808 with *P *< 0.05; *r *between age and LOS was 0.135 with *P *< 0.05. So, all the variables were put into ANN and decision tree models follows.

**Table 1:** Results of univariate analyses on the total charge

variables		t/F	variables		t/F
Sex:			Insurance:		
men	11957.63 ± 8733.77	1.839	private	13696.08 ± 10388.73	1.606
women	13066.63 ± 10277.53		public	12372.86 ± 9520.96	
Marriage:			Dwelling:		
no	12473.81 ± 9708.01	6.164*	county	13361.71 ± 10629.09	
yes	19806.29 ± 5137.35		Suburban	11384.77 ± 9114.22	7.778*
Operation:			urban	14022.70 ± 9665.03	
Without	3292.30 ± 2137.66	54.460*			
With	19666.21 ± 6765.87		Operation type:		
Non-chemotherapy	14268.60 ± 10227.06	7.798*	SG	19526.35 ± 6246.40	
Chemotherapy	9759.44 ± 7930.49		TG	19954.17 ± 5404.27	
Non-radiotherapy	12693.31 ± 9688.01	32.181*	GEL	22856.43 ± 2105.23	237.529*
Radiotherapy	2833.26 ± 11.823		PO	13302.72 ± 2483.79	
			others	20703.42 ± 12433.20	

*Abbr*: SG: subtotal gastrectomy; TG: total gastrectomy; GEL: gastrectomy with extended lymphadenectomy; PO: palliviate operation.

*: *P *< 0.05

### 2. ANN model

It was found that the importance of these factors on the hospital charge on gastric cancer patients were different after the fitting of ANN model. Based on the sensitivity analysis, the most important 5 factors were LOS (0.248396) followed by operation or not (0.196829), emergency treatments (0.176399), type of operations (0.163112) and times of being rescued (0.141685) respectively (The numbers in the brackets represented sensitivities). The linear correlation coefficient and accuracy of the test dataset in the ANN model were 0.987 and 98.35% respectively which were greater than those of training dataset in the ANN model (being 0.955 and 97.418%). Mean absolute error of the test dataset was 1162.279, which was smaller compared with 1819.197 of the training dataset. All these comparisons showed that the prediction ability of the ANN model on the hospital charge of gastric cancer patients was better than its adaptive capacity.

### 3. Decision tree model

The decision tree consisted of 10 leaf nodes and 5 layers. The details of the classification rules were listed in table 2. The linear correlation coefficient of the training dataset of decision tree model was 0.866, which was larger than 0.806 of the test dataset decision tree model. Mean absolute error of the training dataset model was 2782.423, which was smaller than 3424.608 of test dataset model. All these comparisons showed that the adaptive capacity of the decision tree model was better than its prediction ability.

**Table 2:** Classification rules of decision tree of the hospital charge on gastric cancer patients

category	rules	Predictive value(RMB)
A	with operation	19392.655
B	without operation and chemotherapy, LOS< = 3.5 days	1182.421
C	without operation and chemotherapy, 3.5<LOS< = 5.5 days, age< = 42.5 years	1241.267
D	without operation and chemotherapy, LOS>5.5 days, age< = 42.5 years	2081.700
E	without operation and chemotherapy, age>42.5 years, without radiotherapy	2052.962
F	without operation and chemotherapy, age>42.5 years, with radiotherapy	2833.263
G	without operation, with chemotherapy, LOS< = 8.5 days, age< = 59.5 years	4249.538
H	without operation, with chemotherapy, LOS< = 8.5 days, 59.5 year<age< = 70.5 years	3578.098
I	without operation, with chemotherapy, LOS< = 8.5 days, age>70.5 year	5968.295
J	without operation, with chemotherapy, LOS>8.5 days	8175.892

### 4. Comparison of the two predictive models

The ANN model and decision tree model of the two datasets (training dataset and test dataset) were compared in terms of mean absolute error and linear correlation coefficient in table 3.

**Table 3:** Comparison of ANN model with decision tree model of the hospital charge on gastric cancer patients

Indexes	ANN		Decision tree	
	Training dataset	test dataset	Training dataset	test dataset
Minimum error	-10878.152	-6048.823	-12680.355	-11754.598
Maximum error	15072.62	5971.064	36086.455	50410.532
Mean error	81.358	-2.027	0.0	0.0
Mean absolute error	1819.197	1162.279	2782.423	3424.608
Standard deviation	2765.489	1646.117	4689.292	6102.192
Linear correlation coefficient	0.955	0.987	0.866	0.806
Accuracy	97.418%	98.35%	----	----

## Discussion

In our study, the ANN model and decision tree model of two datasets were compared in terms of mean absolute errors and linear correlation coefficients. It was found that: for both the training and the test datasets, mean absolute errors of ANN model were lower than those of decision tree model and linear correlation coefficients of the former were higher than those of the latter. The predictive ability and adaptive capacity of ANN model were better than those of decision tree model.

Seung-Mi Lee [14] conducted a similar study, in which the prediction efficients of ANN and decision tree on the hospital charge on colon cancer patients in Korean were compared. Lee compared ANN model and decision tree model using training and test datasets generated from two groups of patients with different payment schemes, i.e. payment by patients themselves and by public insurance. Lee's study revealed that the prediction efficient of ANN model was superior to the decision tree model for patients whose hospital charge was paid by public insurance; but it was difficult to measure which model was better than the other for patients who paid the hospital charge by themselves. We think that the reason may be as follows: if hospital charges is to be covered by public insurance, the treatment could be performed based on needs of progress of the disease and thus lead to more "reasonable" hospital charges; on the contrary, if hospital charge is to be paid by patients themselves, the treatment could be interfered by some subject factors of the patients, and thus lead to uneven compositions of hospital charges. Given these Lee still concluded that the prediction efficient of ANN model for the hospital charge on colon cancer patients was superior to the decision tree model.

In our study, 18.85% of the patients paid the charges by themselves and 81.15%, by public insurance. Payment system was not found to have significant effects on the hospital charges on gastric cancer patients in the fitness of the two models. This is inconsistent with the report by Lim JH [20]. So we did not compare the two models for these two different groups of patients.

It was found, from the results of the fitness of ANN model, that length of stay was the most important factor on the hospital charge on gastric cancer patients. One explanation for this may be that inpatients' medication was not interrupted and bed charge was inevitable; at the same time, the major components of the hospital charge were just medicine and treatment charges. Furthermore, operation, emergency treatments, type of operation and times of being rescued were also important factors on the hospital charge of gastric cancer patients.

In spite that the prediction efficient of decision tree model was found inferior to ANN model in our study, the method provides directly visible classification rules. Acting on these classification rules, the hospital charge on gastric cancer patients could be easily controlled so as to avoid the phenomenon of inappropriate services.

Additionally we should consider that, as a "black box", BP in ANN would hide some effects of any possible interactions, which was the limitation of this method.

Of course, the gastric cancer patients were drawn only from one hospital in Anhui province of China. If more information of the hospital charge of gastric cancer patients was collected from every region of China, the results should be more reliable. Moreover, given the arithmetic traits of ANN and decision tree models, the choice of predictive models could be performed depending on different research emphasis; or the two models could be used in combination.

## Conclusion

ANN model performed better in predicting hospital charges of gastric cancer patients of China than did decision tree model.

## Competing interests

The authors declare that they have no competing interests.

## Authors' contributions

WJ carried out the meta-analysis studies, participated in the sequence alignment, drafted the manuscript and performed the statistical analysis. LM participated in its design and coordination. HYT participated in the design of the study and the sequence alignment. ZY participated in the design of the study and performed the statistical analysis.

## Pre-publication history

The pre-publication history for this paper can be accessed here:

http://www.biomedcentral.com/1472-6963/9/161/prepub

## References

[B1] Ferlay J Bray F Pisani P Parkin DMcancer incidence, mortality and prevalence worldwide 2001 version 1.0. IARC Cancer Base No 5. Lyon: IARC Press

[B2] Townsend CM Jr Beauchamp RD Evers BM Mattox KL Sabiston Textbook of Surgery 2008 18 Saunders, An Imprinter of Elsevier. Philadelphia

[B3] Beekmann SE Diekema DJ Chapin KC Doern GV Effects of rapid detection of bloodstream infections on length of hospitalization and hospital charges J Clin Microbiol 2003 41 3119 3125 10.1128/JCM.41.7.3119-3125.2003 12843051 PMC165359

[B4] Chang KC Tseng MC Costs of acute care of first-ever ischemic stroke in Taiwan Stroke 2003 34 e219 221 10.1161/01.STR.0000095565.12945.18 14551400

[B5] Rosenman M Madsen K Hui S Breitfeld PP Modeling administrative outcomes in fever and neutropenia: clinical variables significantly influence length of stay and hospital charges J Pediatr Hematol Oncol 2002 24 263 268 10.1097/00043426-200205000-00009 11972093

[B6] Patel JL Goyal RK Applications of artificial neural networks in medical science Curr Clin Pharmacol 2007 2 217 226 10.2174/157488407781668811 18690868

[B7] Kelly DG Stability in contractive nonlinear neural networks IEEE Trans Biomed Eng 1990 37 231 242 10.1109/10.52325 2328998

[B8] Dayhoff JE DeLeo JM Artificial neural networks: opening the black box Cancer 2001 91 8 Suppl 1615 1635 10.1002/1097-0142(20010415)91:8+<1615::AID-CNCR1175>3.0.CO;2-L 11309760

[B9] Marshall AH McClean SI Shapcott CM Millard PH Modelling patient duration of stay to facilitate resource management of geriatric hospitals Health Care Manag Sci 2002 5 313 319 10.1023/A:1020394525938 12437281

[B10] Walczak S Scharf JE Transfusion cost containment for abdominal surgery with neural networks Neural Processing Letters 2000 11 229 238 10.1023/A:1009667711423

[B11] Burke HB Goodman PH Rosen DB Henson DE Weinstein JN Harrell FE Jr Marks JR Winchester DP Bostwick DG Artificial neural networks improve the accuracy of cancer survival prediction Cancer 1997 79 857 862 10.1002/(SICI)1097-0142(19970215)79:4<857::AID-CNCR24>3.0.CO;2-Y 9024725

[B12] Stephan C Büker N Cammann H Artificial neural network (ANN) velocity better identifies benign prostatic hyperplasia but not prostate cancer compared with PSA velocity BMC Urology 2008 8 10 10.1186/1471-2490-8-10 18764937 PMC2543033

[B13] Sargent DJ Comparison of artificial neural networks with other statistical approaches: results from medical data sets Cancer 2001 91 Suppl 8 1636 1642 10.1002/1097-0142(20010415)91:8+<1636::AID-CNCR1176>3.0.CO;2-D 11309761

[B14] Lee Seung-Mi Kang Jin-Oh Suh Yong-Moo Comparison of hospital charge prediction models for colorectal cancer patients:neural network vs.decision tree models J Korean Med Sci 2004 19 677 681 15483343 10.3346/jkms.2004.19.5.677 PMC2816330

[B15] Biddiss EA Chau TT Multivariate prediction of upper limb prosthesis acceptance or rejection Disabil Rehabil Assist Technol 2008 10 1 12 19238719 10.1080/17483100701869826

[B16] Gortzis LG Sakellaropoulos F Ilias I Stamoulis K Dimopoulou I Predicting ICU survival: a meta-level approach BMC Health Serv Res 2008 8 157 10.1186/1472-6963-8-157 18655727 PMC2516515

[B17] Wegrzyn JL Drudge TM Valafar F Hook V Bioinformatic analyses of mammalian 5'-UTR sequence properties of mRNAs predicts alternative translation initiation sites BMC Bioinformatics 2008 9 232 10.1186/1471-2105-9-232 18466625 PMC2396638

[B18] Moseley LG Mead DM Predicting who will drop out of nursing courses: a machine learning exercise Nurse Educ Today 2008 28 469 475 10.1016/j.nedt.2007.07.012 17920163

[B19] Williams JR The Declaration of Helsinki and public health Bulletin of the World Health Organization 2008 86 650 651 10.2471/BLT.08.050955 18797627 PMC2649471

[B20] Lim JH Choi KS Kim SG Park EC Park JH Effects of private health insurance on health care utilization and expenditures in Korean cancer patients: focused on 5 major cancers in one cancer center J Prev Med Pub Health 2007 40 329 335 10.3961/jpmph.2007.40.4.329 17693737

